# Systematic review and meta-analysis of selected toxicities of approved *ALK* inhibitors in metastatic non-small cell lung cancer

**DOI:** 10.18632/oncotarget.25154

**Published:** 2018-04-24

**Authors:** Rubens Barros Costa, Ricardo L.B. Costa, Sarah M. Talamantes, Jason B. Kaplan, Manali A. Bhave, Alfred Rademaker, Corinne Miller, Benedito A. Carneiro, Devalingam Mahalingam, Young Kwang Chae

**Affiliations:** ^1^ Developmental Therapeutics Program, Northwestern University, Chicago, IL, USA; ^2^ Department of Breast Oncology, Lee Moffitt Cancer Center, Tampa, USA; ^3^ Northwestern University, Feinberg School of Medicine, Chicago, IL, USA; ^4^ Northwestern University, Department of Preventive Medicine, Chicago, IL, USA; ^5^ Galter Health Sciences Library, Northwestern University, Chicago, IL, USA; ^6^ Life Span Cancer Institute, Providence, RI, USA

**Keywords:** crizotinib, ceritinib, alectinib, brigatinib, anaplastic lymphoma kinase

## Abstract

**Introduction:**

Anaplastic lymphoma kinase (*ALK*) inhibitors are the mainstay treatment for patients with non-small cell lung carcinoma (NSCLC) harboring a rearrangement of the *ALK* gene or the *ROS1* oncogenes. With the recent publication of pivotal trials leading to the approval of these compounds in different indications, their toxicity profile warrants an update.

**Materials and Methods:**

A systematic literature search was performed in July 2017. Studies evaluating US FDA approved doses of one of the following *ALK* inhibitors: Crizotinib, Ceritinib, Alectinib or Brigatinib as monotherapy were included. Data were analyzed using random effects meta-analysis for absolute risks (AR), study heterogeneity, publication bias and differences among treatments.

**Results:**

Fifteen trials with a total of 2,005 patients with evaluable toxicity data were included in this report. There was significant heterogeneity amongst different studies. The pooled AR of death and severe adverse events were 0.5% and 34.5%, respectively. Grade 3/4 nausea, vomiting, diarrhea, and constipation were uncommon: 2.6%, 2.5%, 2.7%, 1.2%, respectively.

**Conclusions:**

*ALK* inhibitors have an acceptable safety profile with a low risk of treatment-related deaths. Important differences in toxicity profile were detected amongst the different drugs.

## INTRODUCTION

Lung cancer is the most common cause of cancer death in men and the second leading cause of cancer death in women worldwide. Non-small cell lung carcinoma (NSCLC) harboring rearrangements of the anaplastic lymphoma kinase (*ALK*) gene and the *ROS1* oncogene constitute a unique molecular subgroup of this patient population. They comprise approximately 5% and 1% of all the NSCLC cases, respectively [1, 2]. ALK inhibitors may represent an important potential treatment in this setting.

The early signal of efficacy noted in this class of agents led regulatory agencies to fast track clinical development from Phase 1 dose-finding studies straight to phase 3 trials, resulting in less toxicity data than would have been attained otherwise [3–5].

Crizotinib was the first-in-class *ALK* inhibitor developed and evaluated in patients with NSCLC harboring *ALK* rearrangements. Utilizing medicinal chemistry and rational design, different groups have then been successful in the synthesis of novel, selective and potent *ALK* inhibitors with acceptable and consistent pharmacokinetic and pharmacodynamics profiles displaying strong *in vivo* efficacy in *ALK*-positive NSCLC xenograft models at well-tolerated doses. This has led to further development of these drugs [6]. Differences in the chemical structures amongst the *ALK* inhibitors may result in different toxicity profiles and efficacy [7].

Multiple *ALK* inhibitors including Crizotinib, Ceritinib, Alectinib, and Brigatinib have shown efficacy in the subset of *ALK*-rearranged NSCLC in the first and subsequent lines of therapy [3–5, 8–16]. Crizotinib was compared to chemotherapy in previously treated patients, with a median Progression- Free Survival (PFS) of 7.7 months in the Crizotinib group and 3.0 months in the chemotherapy group (95% confidence interval [CI], 0.37 to 0.64; *P* < 0.001). Overall response rates (ORR) were higher in the Crizotinib group than in the chemotherapy group: 65% with Crizotinib versus 20% with chemotherapy [4]. In the treatment-naïve setting, PFS was significantly longer with Crizotinib than with chemotherapy (10.9 months vs. 7.0 months, 95% CI, 0.35 to 0.60; *P* < 0.001). The ORR was significantly higher with Crizotinib than with chemotherapy (74% versus 45%, (*P* < 0.001)) [3]. A phase 3 trial compared Ceritinib to standard chemotherapy in patients who progressed following Crizotinib and a platinum-based doublet. Ceritinib showed a significant improvement in median PFS compared to chemotherapy (5·4 months for Ceritinib compared to 1·6 months for chemotherapy). ORR were 7% for the chemotherapy group as compared with 39% for the Ceritinib group, indicating that *ALK* rearrangements are predictive of benefit to targeted therapy after progression on first line treatment [11]. Resistance mechanisms including mutation of the kinase domain, amplification of the gene copy number, bypass signaling, transformation to small cell lung cancer, have been previously described [17].

The kinase domains of both *ALK* and *ROS1* share significant amino acid homology within the ATP-binding sites [18]. Pre-clinical data support the use of *ALK* inhibitors as a potential target for *ROS1* mutation in NSCLC. For instance, Crizotinib has been shown to induce anti-proliferative activity, inhibit putative downstream targets, and induce apoptosis in *ALK* and *ROS1*-translocated cell lines [19]. Crizotinib showed a median PFS of 19.2 months and ORR of 72% in the expansion cohort of the pivotal phase I trial of patients with NSCLC with tumors harboring a *ROS1* fusion. In a phase 2 trial, Ceritinib showed a median PFS of 9.3 months for all patients and 19.3 months for Crizotinib-naive patients with an ORR of 62% [20, 21]. In a retrospective analysis of *ROS1* fusion-positive patients, Crizotinib showed a higher overall response rate (ORR); disease control rate (DCR) and longer PFS (PFS) compared to pemetrexed and non-pemetrexed based chemotherapy. ORR, DCR, and PFS were 80%, 90.0%, and 294 days, respectively, for Crizotinib, 40.8%, 71.4%, and 179 days, respectively, for pemetrexed chemotherapy, and 25.0%, 47.7%, and 110 days, respectively, for non-pemetrexed chemotherapy. Taken together, these data suggest superior efficacy of the *ALK* inhibitors compared to chemotherapy in this molecularly distinct subgroup of patients [22].

The National Comprehensive Cancer Network guidelines recommend testing for *ALK* rearrangement and *ROS1* fusion for individuals with metastatic NSCLC since *ALK* inhibitors are recommended for the treatment of metastatic NSCLC in the first and second lines settings. Crizotinib is considered the first choice in the treatment of *ROS 1* rearrangement-positive metastatic NSCLC [23].

The purpose of this systematic review and meta-analysis is to update the side effect profile of *ALK* inhibitors in NSCLC with a focus in select adverse events, considering the recent approvals and very recent publication of full manuscripts of respective clinical trials. Recent toxicity data may be used as tool for the selection of ALK inhibitors.

## MATERIALS AND METHODS

### Search strategy

A systematic literature search was performed in July 2017 by a medical librarian in adherence with the Preferred Reporting Items for Systematic Reviews and Meta-Analyses (PRISMA) statement [24, 25]. Subject headings and keywords were used to locate literature in the English language on the use of select *ALK* inhibitors (Crizotinib; Ceritinib; Alectinib; Brigatinib) in Non-Small Cell Lung Cancer in MEDLINE via PubMed 1946- July 2017, EMBASE 1947- July 2017, and Cochrane Library. The full search strategy for PubMed is provided as supplementary data. The database was searched for articles published on or before July 24, 2017. All publication dates were included. Only fully published manuscripts were included in this analysis.

### Selection of trials and data extraction

Inclusion criteria were as follows: 1- Phase 1 expansion-cohort, phase 2, phase 3 or the control arm of a Phase 3 trial using the FDA-approved dose of the particular *ALK* inhibitor (Crizotinib 250 mg twice daily, Ceritinib 750 mg once daily, Alectinib 600 mg twice daily, Brigatinib 180 mg once daily) for the treatment of metastatic *ALK*-rearranged or metastatic/recurrent ROS-rearranged NSCLC; 2- English language. Pediatric or dose-finding Phase I clinical trials were excluded. Studies or study arms that used non-FDA approved doses were also excluded (e.g., Alectinib 300 mg twice daily and/or Brigatinib 90 mg once daily). Other studies using *ALK* inhibitors in earlier development such as Lorlatinib or entrectinib were also excluded. Each publication was reviewed, and in cases of duplicate publication, only the most complete, recent and updated report of a clinical trial was included in this meta-analysis.

For publications meeting inclusion criteria, the following data were extracted: the total number of individuals evaluable for toxicity, number of all grade adverse events (AE), number of grade 3 and 4 AEs, number of deaths related to study drug, and number of discontinuation of treatment due to AEs. Additionally, the number of select grade 3 and 4 AEs were reported [i.e., nausea, vomiting, constipation, diarrhea, fatigue, ILD (interstitial lung disease), QT prolongation]. The relationship between AEs and treatment administration (i.e., treatment-related AEs vs. all-causality) was also documented. The data extraction was performed primarily by the first author (R.B.C.) and subsequently was reviewed by another coauthor (S.M.T.).

### Statistical methods

Meta-analyses were conducted using one-sample proportions to obtain random effects, estimates of toxicity rates and 95% confidence intervals. Heterogeneity was assessed by the *Q* chi square statistic [26]. The percent of total variance due to study heterogeneity was estimated using the I squared (I^2^) statistic. Heterogeneity across treatment types was assessed using the between-group *Q* statistic [27]. Publication bias was evaluated using Egger’s test [28]. Analyses were conducted using Comprehensive Meta-Analysis software package (Comprehensive Meta-Analysis, Version 3.3.070, 2014, Biostatic; Englewood NJ).

## RESULTS

### Study inclusion and characteristics

**Figure 1:**
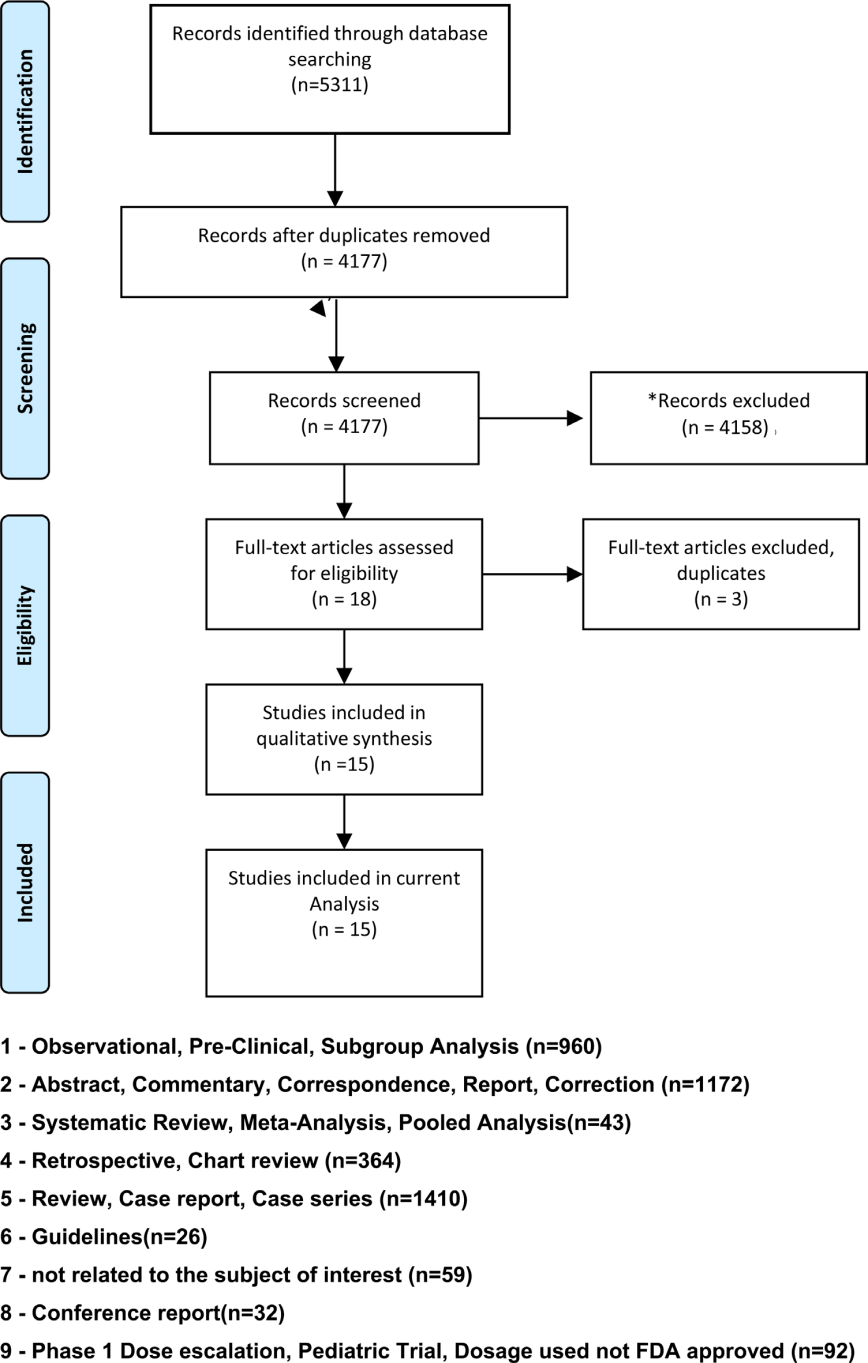
Flow diagram - study selection

Our search strategy yielded 5311 entries through PubMed, EMBASE, and Cochrane combined. 1134 duplicates were initially removed with the deduplication tools in EndNote and Covidence. After examining titles and abstracts, 4158 more entries were excluded. Studies excludedare shown in Figure 1. Fifteen studies met the inclusion criteria and data were extracted. Details of studies selected are described in Table 1. Six studies evaluated Crizotinib; three studies evaluated Alectinib; five studies evaluated Ceritinib; one study evaluated Brigatinib. One of the manuscripts reported the use of Crizotinib for the treatment of *ROS1* positive metastatic NSCLC. A different manuscript reported the use of Ceritinib for *ROS1* positive metastatic and/or recurrent NSCLC. The remainder of the manuscripts used one of the compounds in the treatment of *ALK* positive metastatic and or recurrent NSCLC.

**Table 1:** Characteristics of included trials

Study Author	PMID	Study Phase	Tumor Type	ALK inhibitor dose	Number of patients with ECOG PS ≥ 2	Number of patients evaluable for toxicity	Median follow-up time (months)	NCI CTCAE version
**Kim**	28475456	2	NSCLC- ALK positive	Brigatinib 180 mg QD	9	110	8.3	4
**Peters**	28586279	3	NSCLC- ALK positive	Alectinib 600 mg BID	10	152	18.6	4
**Peters**	28586279	3	NSCLC- ALK positive	Crizotinib 250 mg BID	10	151	17.6	4
**Shaw**	26708155	2	NSCLC- ALK positive	Alectinib 600 mg BID	9	86	9.9	4
**OU**	26598747	2	NSCLC- ALK positive	Alectinib 600 mg BID	13	138	10.96	4
**Kim**	26973324	1(expansion-cohort)	NSCLC- ALK positive	Ceritinib 750 mg QD	28	246	11.1	4
**Crino**	27432917	2	NSCLC- ALK positive	Ceritinib 750 mg QD	20	140	11.3	4
**Shaw**	28602779	3	NSCLC- ALK positive	Ceritinib 750 mg QD	9	115	16.6	4
**Lim**	28520527	2	NSCLC- ROS 1 positive	Ceritinib 750 mg QD	4	32	14	4
**Soria**	28126333	3	NSCLC- ALK positive	Ceritinib 750 mg QD	13	189	19.2	4
**Shaw**	25264305	1(expansion- cohort)	NSCLC- ROS 1 positive	Crizotinib 250 mg BID	1	50	16.4	3
**Solomon**	25470694	3	NSCLC- ALK positive	Crizotinib 250 mg BID	10	171	17.4	4
**Camidge**	22954507	1(expansion –cohort)	NSCLC- ALK positive	Crizotinib 250 mg BID	18	149	16.3	3
**Shaw**	23724913	3	NSCLC- ALK positive	Crizotinib 250 mg BID	16	172	12.2	4
**Hida**	28501140	3	NSCLC-ALK positive	Crizotinib 250 mg BID	2	104	12.2	4

Abbreviations: BID = Twice Daily; ECOG = Eastern Cooperative Group; NCIC CTCAE = National Cancer Institute Common Terminology Criteria for Adverse Events; NSCLC = Non-Small Cell Lung Cancer; PMID = PubMEd ID; QD = Once Daily.

### Description of study participants

A total of 2005 individuals were evaluable for toxicity in all fifteen studies. Only 82 patients had tumors harboring *ROS1* aberrations. The median age at study entry was 54.6 years. All of the patients had either stage IIIB or stage IV (*de novo* vs. recurrent) disease. Approximately 8.5% of the patients accrued in these trials had ECOG Performance Status ≥ 2.

### Study-to-study heterogeneity and publication bias

There was significant inter-study heterogeneity for most toxicities. Heterogeneity I^2^ statistics for any grade, any serious, grade 3-4 AE were as follows: 36.5%; 82.6%, and 96.1%, respectively. Egger’s test showed publication bias for many toxicities.

### Number of all-grade, grade 3/4 and serious AEs, number of treatment related deaths, and number of patients who discontinued treatment due to toxicity across different studies

Adverse events were classified and graded according to the Common Terminology Criteria for Adverse Events, version 3.0 or version 4.0. Safety data across the studies were not reported in a uniform fashion. Data were missing for some of the endpoint analyzed in this report. Five out the fifteen studies did not report the number of total AEs. Seven did not report the total number of grade 3 and 4 AEs. Six did not reports serious AE. One study reported an aggregate of grade 3-5 toxicities. Thirteen studies reported the number of patients who experienced an AE. Data were missing for any-grade, grade 3 and 4, serious AEs for 2 studies which reported the total number of AEs. The other studies reported the number of patients who experienced an AE.

Absolute toxicity rates and results of the random effects meta-analysis of each toxicity rate are summarized in Table 2. Toxicity rates for any AE, any serious AE, and grade 3/4 AE were as follows: 98.4% (95% CI, 96.9–99.2), 34.5% (95% CI, 28.1–41.6), and 64% (95% CI, 47.1–78), respectively. All fifteen studies reported the number of patients who discontinued treatment due to toxicities. The pooled AR of discontinuation for all four *ALK* inhibitors (Crizotinib, Alectinib, Ceritinib, Brigatinib) due to toxicity was approximately 8.2%. All 15 studies described reported treatment-related death rates. Only, 10 treatment-related casualties were reported and were considered deaths due to the use of one of the drugs by the investigators. Causes of death included the following: 1 case of bowel perforation, 1 case of unspecified hemorrhage, 1 case of cardiac arrhythmia, 4 cases of interstitial lung disease/pneumonitis; 1 case of multiorgan failure; 2 cases of treatment-related deaths not otherwise specified. For all the of studies, the authors made an effort to make a distinction between grade 5 toxicity and treatment-related deaths. Treatment-related deaths represented less than 0.5% of the population evaluable for toxicity in this report.

**Table 2:** Meta analysis summary of adverse events

AE	Number of studies	Number of evaluable patients	Number of patients with AE	Random effects toxicity rate (%)	Random effects 95% confidence interval	Heterogeneity *p*-value	I^2^
**Any AE**	10	1450	1435	98.4	96.9–99.2	0.12	36.5
**Any Serious AE**	9	1198	430	34.5	28.1–41.6	< 0.001	82.6
**Any Grade 3/4 AE**	8	1147	751	64.0	47.1–78.0	< 0.001	96.1
**Diarrhea**	15	2005	1133	54.4	40.5–67.6	< 0.001	96.5
**Diarrhea 3/4**	15	2005	49	2.6	1.6–4.2	0.013	50.7
**Nausea**	15	2005	1087	51.5	29.5–63.3	< 0.001	95.8
**Nausea 3/4**	15	2005	53	2.5	1.6–4.1	0.003	56.9
**Vomiting**	15	2005	852	38.3	29.1–48.3	< 0.001	94.3
**Vomiting 3/4**	15	2005	51	2.7	1.8–4.2	0.025	46.4
**Constipation**	14	1973	618	31.0	26.5–35.8	< 0.001	79.7
**Constipation 3/4**	14	1973	12	1.2	0.7–1.9	0.68	0.0
**Fatigue**	14	1901	533	27.2	22.9–31.9	< 0.001	78.8
**Fatigue 3/4**	14	1901	54	3.2	2.0–5.0	0.004	57.7
**ALT**	12	1552	502	28.2	19.8–38.3	< 0.001	93.5
**ALT 3/4**	12	1552	239	11.1	7.1–16.9	< 0.001	89.4
**AST**	13	1662	447	24.8	18.3–32.5	< 0.001	90.7
**AST 3/4**	13	1662	125	6.8	4.6–9.9	< 0.001	74.5
**QTc**	5	477	40	8.6	4.9–14.8	0.04	60.1
**QTc 3/4**	11	1558	28	2.1	1.2–3.6	0.061	43.4
**ILD**	9	1255	26	2.2	1.2–4.0	0.04	50.5
**ILD 3/4**	10	1346	17	1.9	1.2–3.0	0.54	0

Abbreviations: AE = Adverse Event; ALT = Alanine Aminotransferase; AST = Aspartate Aminotransferase; ILD = Interstial Lung Disease.

^*^Meta-analyses were conducted using one-sample proportions to obtain random effects, estimates of toxicity rates and 95% confidence intervals. Heterogeneity was assessed by the *Q* chi square statistic. *p* ≤ 0.05 was considered statistically significant.

The percent of total variance for study heterogeneity was estimated using the I squared (I^2^) statistic. Analyses were conducted using Comprehensive Meta-Analysis software package (Comprehensive Meta-Analysis, Version 3.3.070, 2014, Biostatic; Englewood NJ).

### Number of all-grade and grade 3/4 gastro intestinal (GI) and other selected toxicities

The four most common GI all-grade toxicities were diarrhea, nausea, vomiting, and constipation, respectively. They were consistently reported across all studies. Only one study did not report the rates of constipation. Estimated AR for all grade diarrhea, nausea, vomiting and constipation were: diarrhea 54% (95% CI, 41–68), nausea 52% (95% CI, 40–63), vomiting 38% (95% CI, 29–48) and constipation 32% (95% CI, 27–36). The risk for grade 3/4 diarrhea, nausea, vomiting and constipation were as follows: diarrhea 2.6% (95% CI, 2–4); nausea 2.5% (95% CI, 2–4); vomiting 2.7% (95% CI, 2–4); constipation 1.2% (95% CI, 1–2). The estimated AR for any visual disturbance was of 43.5% in only 4 of the crizotinib studies. One case of visual disturbance occurred with the use of ceritinib and no cases occurred with the use of Alectinib or Brigatinib. Grade 3/4 QTcB prolongation was reported in 11 of the studies included in the analysis, with a random-effect pooled risk of 2.1% (95% CI, 1–4). Grade 3/4 ILD was reported in 9 studies occurring in a total of seventeen individuals. Grade 3/4 ALT and AST elevations were infrequent events. Grade 3/4 fatigue was reported in 14 of the 15 studies, with a random-effect pooled AR of 3.2% (95% CI, 2–5).

### All grade, grade 3/4, serious AE toxicity rates across different treatment groups

Differences in AR of any grade, grade 3/4, serious AE were detected amongst different treatment groups (Table 3). There were also differences amongst the selected AEs in relation to GI toxicity including nausea, vomiting and diarrhea. Ceritinib was associated with a high rate of nausea, vomiting and diarrhea with a low chance of Grade 3/4 toxicity. QTcB prolongation was rare. A more detailed description of risk of selected toxicities according to treatment group is shown in Table 3.

**Table 3:** Adverse events (%) by treatment drug (included trials)

AE	Alectinib	Brigatinib	Ceritinib	Crizotinib	*p*-value^*^
**Any AE (%)**	96.7	ND	99.6	97.7	0.022
**Any Serious AE (%)**	21.6	ND	44.9	31.1	< 0.001
**Any Grade 3/4 AE (%)**	ND	ND	75.3	43.4	0.009
**Diarrhea (%)**	13.7	38.2	81.2	56.0	< 0.001
**Diarrhea 3/4 (%)**	0.6	0.5	5.6	1.7	< 0.001
**Nausea (%)**	15.3	40.0	73.9	55.3	< 0.001
**Nausea 3/4 (%)**	0.5	0.9	5.7	1.8	< 0.001
**Vomiting (%)**	9.8	22.7	60.4	43.9	< 0.001
**Vomiting 3/4 (%)**	0.6	0.5	5.2	2.0	< 0.001
**Constipation (%)**	34.1	15.5	24.3	37.1	< 0.001
**Constipation 3/4 (%)**	0.4	0.5	0.8	1.5	0.37
**Fatigue (%)**	25.7	27.3	34.5	21.7	0.039
**Fatigue 3/4 (%)**	1.0	0.5	6.0	2.1	< 0.001
**ALT (%)**	14.3	ND	46.9	21.8	< 0.001
**ALT 3/4 (%)**	4.0	ND	22.8	9.1	< 0.001
**AST (%)**	15.0	14.5	38.8	21.0	< 0.001
**AST 3/4 (%)**	3.9	0.5	11.4	5.7	0.007
**QT (%)**	1.2	ND	9.0	14.4	0.025
**QT 3/4 (%)**	0.8	ND	0.9	3.9	0.003
**ILD (%)**	0.6	ND	2.4	2.4	0.610
**ILD 3/4 (%)**	0.4	ND	2.1	2.0	0.310

Abbreviations: AE = Adverse Event; ALT = Alanine Aminotransferase; AST = Aspartate Aminotransferase; ILD = Interstial Lung Disease; ND = No data available;

^*^Heterogeneity across treatment was assessed using the between-group *Q* statistic, *p* ≤ 0.05 was considered statistically significant.

## DISCUSSION

In this analysis, all four of the approved *ALK* inhibitors have shown acceptable toxicity profiles with the majority of AEs being either grade 1 or 2. Fewer than 0.5% of the pooled population with evaluable toxicity were noted to have treatment-related deaths (*n* = 10). The combined rate of discontinuation for all of the 4 drugs was approximately 8.2%.

Furthermore, we analyzed the AR of selected AEs between studies and amongst the 4 US FDA-approved *ALK* inhibitors. It is important to highlight a few of these toxicities including, nausea, vomiting, constipation, and diarrhea, as they were frequent (predominantly grade 1 or 2). The risk of grade 3/4 nausea, vomiting, diarrhea, and constipation were 2.5%, 2.7%, 2.6%, and 1.2% respectively. They were consistently reported across almost all of the 15 reports included in this analysis. The AR for all-grade fatigue was 27.2% with an AR for grade 3/4 fatigue of 3.2%. Despite being considered significant toxicities, all-grade and grade 3/4 QTcB prolongation and ILD were only described in five and nine out of the 15 studies, respectively. The risk for grade 3/4 QTcB prolongation was 2.1%. These two rates are in line with a recent meta-analysis that showed incidences of high-grade ILD and QTcB prolongation of 2.5% and 2.8%, respectively [29].

Important differences were detected amongst the different *ALK* inhibitors likely due to off-target effects with these drugs. Ceritinib had a high AR of GI toxicity, namely diarrhea, nausea, and vomiting. A plausible explanation is that Ceritinib inhibits the activity of insulin growth factor receptor (IGFR), which is expressed in cells alongside the GI tract in pre-clinical models [6, 7]. Early trials with IGFR inhibitors such as cixutumumab did report GI toxicities such as abdominal pain, nausea, vomiting, diarrhea in different dose escalation cohorts [30, 31]. In preclinical models, EGFR inhibition in repairing airway epithelial cells modulated significant expression of genes involved in the airway microenvironment, prolonged inflammation, and potentiated acute lung injury [32]. Therefore, this may be a plausible explanation for pneumonitis in the early studies with Brigatinib as it inhibits EGFR kinases [33]. Experimental models suggest that ALK inhibitors might target retinal ganglion cells affecting response to light. The drug potency on the these responses might be responsible for the difference in the frequencies of visual disturbances between crizotinib and alectinib [34]. Crizotinib, first-in-class amongst the *ALK* and ROS inhibitors, is still a very effective and safe option for the treatment of patients with *ALK* positive metastatic NSCLC, a condition associated with a poor prognosis and for which limited therapeutic options exist. In a recent meta-analysis, diarrhea, nausea, vomiting, peripheral edema, and constipation were amongst the most common side effects related to Crizotinib [35]. The overall rate of SAEs with Crizotinib was 19.9% in another systematic review [36]. Grade 3 to 5 adverse events occurred at a rate of 50% with Crizotinib versus 41% with Alectinib in the Alex study [14]. In keeping with these reports, the AR of serious and grade 3/4 AEs were 31% and 43%. Nausea, vomiting, and diarrhea were very common in our analysis.

Alectinib is now considered the preferred first-line agent for *ALK* positive metastatic NSCLC patients. A recent pooled analysis from the two pivotal phase 2 trials using Alectinib 600 mg twice daily again confirmed an acceptable safety profile with continued follow-up. Grade 3 or higher AEs occurred at a rate of 40%. The rate of discontinuation of treatment due to AEs was 6%. Dose interruption and/or modification occurred at a rate of 33%. In this analysis, the AR of serious AE was approximately 22%. The AR for Grade 3/4 diarrhea, nausea, vomiting and constipation was below 1% for any of these AEs [36].

Ceritinib may also be used in the first or subsequent lines of therapy for *ALK* positive metastatic NSCLC. In preclinical models, it has been shown to be a more potent *ALK* inhibitor than Crizotinib [37]. However, it has not been directly compared to the first-in-class compound in the first-line or subsequent-line settings. No phase 3 randomized controlled trials comparing these two drugs are being planned or conducted. In this analysis, Ceritinib stands out as having the highest AR of GI toxicities which may hamper its usage in clinical practice. Therefore, it would be important to conceive a strategy to mitigate GI toxicity given its proven efficacy. Results of part 1 of the phase 1 ASCEND 8 trial suggest that Ceritinib may be taken at lower doses (e.g., 450 mg daily, 600 mg daily) with a low-fat meal. At steady state, the 450-mg dose with food demonstrated comparable pharmacokinetics (PK) as assessed by peak concentration of the drug in plasma and area under the curve from 0 to 24 hours. The 600-mg dose with food demonstrated approximately 25% higher PK. The 450-mg dose with food was associated with a lower proportion of patients with GI toxicities: diarrhea, nausea, and vomiting. There were no grade 3 or 4 AEs, study drug discontinuations, or serious AEs due to GI toxicities. Efficacy and long-term safety results will be available once part 2 is concluded [38]. If confirmed, physicians along with patients may feel more confident about the safety of Ceritinib with this strategy.

Brigatinib is another potent *ALK* inhibitor that has demonstrated activity against many *ALK* domain mutations giving rise to resistance to other drugs in the same class. Pre-clinical data have shown increased potency of Brigatinib as compared to Crizotinib [7]. It has been shown to have promising systemic and intracranial activity and an acceptable safety profile for the *ALK* rearranged NSCLC subset [16, 39]. Early pulmonary AEs occurred at 90 mg with no further events occurring after escalation to 180 mg. Therefore, a lead-in dose of 90 mg once daily for 1 week before escalation to 180 mg once daily was chosen for further development [39]. A Phase 3 global clinical Trial (ALTA1L) comparing Crizotinib and Brigatinib in the setting *ALK*-TKI naïve metastatic is ongoing and currently accruing patients (NCT02737501).

The lack of access to individual patient data is the major limitation of this study and does not allow for exploration of correlations between patient characteristics and risk of toxicities. Data on adverse events were missing for several reports. The lack of consistency on how AEs are reported makes it difficult to compare AEs amongst the different studies. For example, in the ALEX study, grade ≥ 3 events were grouped as grade 3-5 events in contrast to the other studies in this analysis where grade ≥ 3 events were grouped as grade 3-4 events. The JALEX trial used a dose of Alectinib of 300 mg Twice Daily and therefore, data from that arm was excluded from our report. The phase I dose finding trial and the 90-mg dose of other studies using Brigatinib were also excluded from this analysis. The only arm used here was the 180-mg arm for Brigatinib with a lead in dose schedule of 90 mg daily for 7 days prior to the approved dose. This may have contributed to a low rate of ILD reported in this analysis.

In the early phase trials, responses were seen at lower doses of *ALK* inhibitors. Shaw et al showed an AR of Grade 3/4 AE of 20% in patients taking 5-300 mg of Ceritinib daily. There was no case of Grade3/4 diarrhea, nausea or vomiting. Partial responses were confirmed in two out of eight patients in that cohort [40]. Grade 3 AEs were reported in 37% of patients in a phase I-II trial conducted in Japan in patients using Alectinib 300 mg twice daily. No grade 4 AEs or deaths were reported. No cases of grade 3 nausea, diarrhea, or vomiting were reported [41]. Thus, using lower doses of an *ALK* inhibitor may mitigate toxicity without compromising efficacy.

## CONCLUSIONS

*ALK* inhibitors have an acceptable safety profile with a low risk of treatment-related deaths. Important differences in toxicity profile were detected amongst the different *ALK* inhibitors. Physicians have several options for the treatment of patients with this distinct molecular subgroup of NSCLC.
